# Impact of traffic variability on geographic accessibility to 24/7 emergency healthcare for the urban poor: A GIS study in Dhaka, Bangladesh

**DOI:** 10.1371/journal.pone.0222488

**Published:** 2019-09-16

**Authors:** Shakil Ahmed, Alayne M. Adams, Rubana Islam, Shaikh Mehdi Hasan, Rocco Panciera

**Affiliations:** Health Systems and Population Studies Division, icddr,b, Dhaka, Bangladesh; University of Utah, UNITED STATES

## Abstract

Ensuring access to healthcare in emergency health situations is a persistent concern for health system planners. Emergency services, including critical care units for severe burns and coronary events, are amongst those for which travel time is the most crucial, potentially making a difference between life and death. Although it is generally assumed that access to healthcare is not an issue in densely populated urban areas due to short distances, we prove otherwise by applying improved methods of assessing accessibility to emergency services by the urban poor that take traffic variability into account. Combining unique data on emergency health service locations, traffic flow variability and informal settlements boundaries, we generated time-cost based service areas to assess the extent to which emergency health services are reachable by urban slum dwellers when realistic traffic conditions and their variability in time are considered. Variability in traffic congestion is found to have significant impact on the measurement of timely access to, and availability of, healthcare services for slum populations. While under moderate traffic conditions all slums in Dhaka City are within 60-minutes travel time from an emergency service, in congested traffic conditions only 63% of the city’s slum population is within 60-minutes reach of most emergency services, and only 32% are within 60-minutes reach of a Burn Unit. Moreover, under congested traffic conditions only 12% of slums in Dhaka City Corporation comply with Bangladesh’s policy guidelines that call for access to 1 health service per 50,000 population for most emergency service types, and not a single slum achieved this target for Burn Units. Emergency Obstetric Care (EmOC) and First Aid & Casualty services provide the best coverage, with nearly 100% of the slum population having timely access within 60-minutes in any traffic condition. Ignoring variability in traffic conditions results in a 3-fold overestimation of geographic coverage and masks intra-urban inequities in accessibility to emergency care, by overestimating geographic accessibility in peripheral areas and underestimating the same for central city areas. The evidence provided can help policy makers and urban planners improve health service delivery for the urban poor. We recommend that taking traffic conditions be taken into account in future GIS-based analysis and planning for healthcare service accessibility in urban areas.

## Background

Accessibility and availability of healthcare are closely bonded concepts; geographic (physical) accessibility addresses the spatial dimension, i.e., physical distance from patient to health service locations, while availability corresponds to the existence of a required type of healthcare [1]. Geographic accessibility is one of the major determinants of (or barriers to) healthcare utilization, and is generally studied in relation to different health service types [2–8]. It is especially crucial for emergency medical services since timely and appropriate emergency care holds the potential to prevent 45% of deaths and 36% of related disability in low- and middle-income countries (LMICs) [9]. Ensuring access to emergency care is a persistent concern for health system planners [10], and minimizing travel time in particular can make a difference between life and death. It is generally accepted that qualified medical assistance during the first hour of trauma decreases the mortality rate substantially [11].

The World Health Organization (WHO) recommends the quantification of geographic accessibility using travel time units, rather than distance, as this more closely captures obstacles associated with geographic separation [12]. This is particularly relevant in urban areas, where even small distances can lead to extended time delays due to congested traffic conditions. Numerous techniques have been proposed to measure the accessibility of health services using Geographic Information Systems (GIS). Such methods include simple provider-to-population ratios [13, 14] and calculations of fixed catchment areas based on straight line buffers [15, 16], network-based catchment area delineation [17], or decaying density functions modeling the decrease in availability of healthcare with distance (kernel density [18]). More advanced methods weight both supply and demand for services within catchment areas (traditional Two-Step Floating Catchment Area– 2SFCA [19], gravity model [20], accessibility index method [21]), and, more recent, techniques incorporating variable decay functions to model the impact of distance on utilization within catchment areas (network-based index [22], modified 2SFCA) [23–25].

Regardless of complexity, all the above methods, largely advanced in developed country settings, tend to disregard variability in traffic conditions by assuming constant and predictable transit times for different road types (i.e., highway, urban/non-urban road, paved/unpaved road) [26–29]. Transit times are generally estimated using available speed limits data and by assigning rules for transit time depending on road type, and rural versus urban settings [7, 16, 30–32]. Models using raster-based cost surfaces based on road network have also been used to calculate accessibility to healthcare in LMIC settings [33–36].

The assumption traditionally made of constant and predictable travel times are inapplicable in growing megacities in LMICs where unregulated traffic conditions significantly affect traffic flow and speed. For example, Dhaka has only around 6% of its total area designated as road where many megacities have around 25% [37]. This, along with high population density [38], is the major factor behind traffic congestion and variability in Dhaka City. The extent to which variable traffic conditions affect the measurement of geographic accessibility to health services, and therefore the assessment of timely accessibility to healthcare, has thus far not received attention in the context of dense urban areas in LMICs.

In this paper, we measure geographic accessibility to emergency services in Dhaka City, Bangladesh, by accounting for information on traffic conditions and variability and their impact on timely access to emergency care services. We make use of the Two-Step Floating Catchment Area (2SFCA) method developed by Luo and Wang [21]. 2SFCA considers both supply of and demand for health services, and has been used extensively to study accessibility to health services such as primary healthcare and cancer [32, 39–45]. To our knowledge this study is among the first to employ 2SFCA method to measure access to emergency healthcare services in LMICs.

To date, the majority of accessibility studies in LMICs have focused on rural areas [1, 29, 46–48]. Only a few have considered the urban scenario [49–52], which is partly a function of the dearth of reliable information on health service locations and their characteristics in urban settings, and the assumption that geographical accessibility is not a major problem in urban areas. Moreover, GIS-based studies in Bangladesh have mostly assessed access to emergency services for obstetric care [48, 52] or primary healthcare in rural areas [49]. Accessibility to other emergency health services such as Burn Units and Intensive Care Units (ICU) in urban settings, have not been considered. This study fills this gap by providing GIS-based evidence of the extent to which emergency health services are reachable when realistic traffic conditions and their variability in time are considered in the megacity of Dhaka. Of particular interest is how these factors mediate against timely access to emergency care by those living in informal or slum settlements. This growing population is persistently deprived of basic rights and services including healthcare [53], yet experience much greater health risks and exposures than non-slum populations. It follows that timely access to emergency healthcare is critical in averting mortality and disability in this vulnerable population.

By combining unique information on emergency health service locations, road networks, traffic flow variability and informal settlements boundaries through GIS analysis of geographic accessibility, this study addresses the following questions:

1. What percentage of urban slum settlements in Dhaka City lack timely access and adequate supply of emergency health services when considering realistic scenarios of traffic congestion?
2. Does accessibility change between different emergency services?
3. Which emergency services provide poorer coverage in Dhaka City?
4. Are there areas of Dhaka City suffering from persistent lack of accessibility to emergency services?
5. What is the impact of variability of traffic conditions when assessing geographic accessibility to healthcare using common methods?

The research presented is novel in two main regards. For the first time it includes consideration of variable traffic conditions in the 2SFCA methods, against the assumption traditionally made of constant and predictable travel times. Secondly, it provides the first assessment of accessibility to emergency services in LMIC urban conditions characterized by intense traffic conditions. This analysis is important in the context of current policy dialogues on urban health in developing countries including Bangladesh, and will demonstrate the need for informed decision-making and planning regarding the equitable placement of emergency facilities in areas of the city where geographic coverage is low, yet need is substantial.

## Materials and methods

This study received clearance from the institutional review board (IRB) of icddr,b, Protocol No. PR-13100 which included a technical review by the Research Review Committee (RRC) and an ethical review by the Ethics Review Committee (ERC). Written informed consent was sought from all the participants involved in the study.

### Study site and population

Dhaka, the capital city of Bangladesh, is the one of fastest growing megacities in the world [54]. Divided administratively into Dhaka North City Corporation (DNCC) and Dhaka South City Corporation (DSCC), its combined surface area is 126.59 sq.km [55, 56], accommodating a population of approximately 8 million [57], although the larger metropolitan area is estimated to be well over 18 million [58]. Dhaka’s population density is nearly 55,000 per sq.km [38], making it one of the most densely populated cities in the world; 4 times higher than Delhi and 10 times greater than Tokyo and Beijing [59]. As the capital city and main economic hub of the country, Dhaka receives at least 1,500 immigrants daily [60], with the majority settling in slums [61]. Around 12% of the Dhaka City Corporation area is currently occupied by slums [62], and about 29% of urban slum dwellers in Bangladesh live in Dhaka [63]. This population is expected to increase at an average rate of 2.72% until 2025 [64].

### Health facilities mapping and road networks

Data on emergency health services locations and characteristics were extracted from the very first comprehensive GIS-based census of urban health facilities conducted by the Health Systems and Population Science Division (HSPSD), icddr,b, Bangladesh. Completed in 2014, the census in Dhaka North and South City Corporation surveyed the geo-locations of all public, private and NGO health facilities and collected facility-specific information on facility type, management entity, services offered, staffing patterns, qualifications and training, opening hours, and basic cost information. The census method employed is described in detail elsewhere [65]. A forty-member team was deployed to collect data and it took around 18 months to complete the census.

Emergency service points (hospitals and clinics) were selected from a database of service types reported by owners or representatives during the health facility census (Table 1). We excluded informal providers, pharmacies, drug shops and doctor chambers given that they tend to provide only limited first aid services. The elevated number of Emergency Obstetric Care (EmOC) and First Aid & Casualty services is noteworthy, due to a significant presence of the private sector. Conversely, Burn Units are only available at 5 service points, all correspondents to public providers.

**Table 1:** Number of emergency service providers in Dhaka City Corporation by management type.

Services	Public		Private		Number of providers
	Direct	Contracted out	For-profit	Not-for-profit	
First Aid & Casualty	30	10	328	58	426
Burn Unit	5	--	--	--	5
Coronary Care Unit (CCU)	8	--	34	4	46
Neonatal Intensive Care Unit (NICU)	4	--	34	3	41
Intensive Care Unit (ICU)	12	--	71	4	87
Emergency Obstetric Care (EmOC)*	12	8	218	24	262

*Both basic and comprehensive

In addition to the census, a comprehensive mapping of the road network was undertaken using Global Positioning System (GPS) tracking. Road network data were collected by tablet computer (tab) equipped with software developed by the icddr,b team, which provided a base map comprised of three layers, namely Google images, data indicating administrative boundaries and road networks collected from Dhaka North and South City Corporation Offices. Field teams walked through the study area, updating base road networks by adding new ones and modifying existing ones, if necessary.

### Modeling geographic accessibility to emergency services

Geographic accessibility to emergency services was calculated by applying the Two-Step Floating Catchment Area (2SFCA) method originally developed by Luo and Wang [21]. Complete details about this method are given elsewhere [32]. In this method, supply (health facilities) and demand (population) for health services are weighted through their respective catchment areas through two main steps. In the first step, supply-to-demand ratios (*R*_*j*_) are calculated for each health facility (*j*) relative to its catchment area, by summing all population locations (*k*) falling within a threshold travel time (*d*_*0*_) from the health facility (j):

$$R_{j}=\frac{S_{j}}{\sum_{k\in \{d_{kj}\leq d_{0}\}}P_{k}}$$ (1)

Here *P*_*k*_ is the population of slum *k* whose centroid falls within the catchment (i.e., *d*_*kj*_ *≤ d*_*0*_), *S*_*j*_ is capacity of health facility at location *j*, and *d*_*kj*_ is the travel time between *k* and *j*. Note that population locations (k) are physical locations where particular population groups concentrate. These can be residences, tract centroids or ZIP centroids, depending on the resolution possible with the data. In this paper, we take as population locations the centroids of the slum area boundary derived from satellite imagery as detailed later on.

In the second step of the 2SFCA method, for each population location *i*, the method searches all facility locations *j* that are within the threshold travel time *d*_*0*_ from slum location *i*, and sums up the supply-to-demand ratios *R*_*j*_ at these locations:

$$A_{i}^{F}=\sum_{j\epsilon \{d_{ij\leq d_{0}}}R_{j}=\sum_{j\epsilon \{d_{ij\leq d_{0}}\}}\left(\frac{S_{j}}{\sum_{k\in \{d_{kj}\leq d_{0}\}}P_{k}}\right)$$ (2)

$A_{i}^{F}$ is the estimated geographic accessibility to health services at location *i*, expressed in terms of availability of healthcare services per unit population, also referred to as the “spatial accessibility score”. The accessibility score ratios so obtained are assigned to the entire area represented by the population point, in our case, each slum. Thus, all population areas, i.e. all slums, have an assigned spatial accessibility score. This can be zero in the case where a slum might be further away than the assumed threshold travel time *d*_*0*_ from any health facility.

In this study we consider facility capacity *S* to be equal to 1, given that the census of urban health facilities did not contain specific information on number of beds or specialists available for special emergency units. This allowed us to express the 2SFCA spatial accessibility score in units of number of emergency services per unit population. In order to categorize slums by different geographic accessibility, we further considered a threshold of emergency services per 50,000 population. There is a guideline for trauma care centre [66] but not regarding the optimum population to be served by each level of facility for emergency services. However, this is of great relevance to the accessibility of trauma care. We therefore use as a reference accessibility score the Bangladesh government target of 1 clinic per 50,000 population for primary healthcare in its Urban Primary Health Care Service Delivery Project (UPHCSDP) that includes emergency obstetric care [67]. Travel times between health facilities and population locations were calculated using ArcGIS 10.2 Network Analyst and the available road network data.

In the following discussion, for simplicity, the term “geographic coverage” will be used to refer to the extent to which slums fall within threshold travel time (*d*_*0*_) from the health facility, and the term “geographic accessibility” is used to denote the spatial accessibility score of the 2SFCA. While geographic coverage provides an indication of whether slum populations are within timely reach of emergency services, geographic accessibility indicates the extent to which this same population has access to an adequate supply of healthcare.

Boundaries of individual slums were obtained from a published study that used high-resolution satellite images verified by ground surveys to map the extent and change in slum areas in Dhaka Metropolitan Area (DMA) from 2006 to 2010 [62]. Slums were defined based on the following criteria: poor housing conditions, high population density and single room occupancy, lack of proper sanitation and water access, high concentration of population with low income, and insecure land tenure. [68]. In this study, the most updated slum map was used (2010) [62]. It should be noted that this dataset did not involve a slum census. As a result, only information on the boundaries and size of each slum cluster was available. In the absence of comprehensive information on population size needed for 2SFCA calculations, we estimated the population of each slum based on slum population density data from another study [69], and calculated the population of each slum by multiplying the slum area by the population density. Centroid of slums for the purpose SFCA calculations were estimated using ArcGIS standard geometric centroid functions.

### Travel time estimate and traffic scenarios

It is common practice in GIS studies of geographic accessibility to assign uniform travel speed across road networks using available information on speed limits, with a differentiation of speed depending on road type and on urban/rural location, and/or population density [7, 29, 32, 39, 49, 70–72]. This approach is not applicable to dense urban areas of Bangladesh, however, where highly congested traffic conditions predominate and where gas-powered 3-wheelers and rickshaws are the more common mode of transportation among urban slum residents [52, 73].

In this paper we employ an innovative approach to calculating geographic accessibility to emergency services that takes into account congested and variable traffic conditions. This was accomplished by using transit speed data from a published traffic survey conducted in Dhaka in 2009 [74] that recorded the distribution of transit speeds for the whole area without considering the road type (in units of minutes per 1 km of road) at 30 different locations in Dhaka every day over a 15 day period. The survey also captured daily variation by monitoring traffic four times during each day: two peak-hour windows (8–9 am, 5–6 pm) and two off-peak windows (12–1 pm, 9–10 pm) (see Table 2).

**Table 2:** Statistics of measured transit times and coefficient of variation in different sampling windows in Dhaka City (from [74]).

Temporal Window	Transit time (minutes per 1 km)	Coefficient of variation
8 am to 9 am	6.2 (mean)	33.5
12 pm to 1 pm	7.6 (mean)	22.8
5 pm to 6 pm	7.9 (mean)	22.5
9 pm to 10 pm	7.2 (mean)	29.4
all windows	4.5 (50th percentile)	
all windows	7.9 (75th percentile)	
all windows	20 (95th percentile)	

Differences in mean travel time comparing morning peak and off-peak hours shown in Table 2 are limited. As noted by the authors of the 2009 traffic study [74], it is difficult to differentiate peak and off-peak in Dhaka City because of severe traffic congestion over the entire day. Concomitantly, the smaller mean travel time in the morning peak (8-9am) is associated with a higher variability of travel time than other times in the day (as shown by the coefficient of variation in Table 2). This implies that, although slightly faster on an average, morning peak hours would be characterized by a higher unpredictability of traffic variability than other times of the day, making our later analysis ever more relevant for this time window.

In virtue of the above, for this study we disregard the variability of mean travel time at different time of the day, which is minimal, and instead focus on the overall variability of travel time across the entire sample [74] in order to estimate different scenarios of traffic congestion. We therefore calculated “geographic accessibility” to emergency services by using three traffic scenarios, estimated using the 50^th^, 75^th^ and 95^th^ percentile of the transit speed distribution including all available days and windows. The scenario using the 50^th^ percentile, or median, transit time, is hereby referred as “moderate” traffic conditions and it is the baseline scenario against which we compare the increasingly congested traffic conditions, captured by the 75^th^ and 95^th^ percentiles of transit time. For each traffic scenario, the corresponding transit time was assumed as uniform across the road network.

We further considered 60-minutes as the threshold travel time for calculation of the catchment areas required by the 2SFCA method. Recognizing that there is no single figure that indicates the best possible window in which emergency services must be delivered as this depends on a patient’s condition and the type of emergency situation experienced, we employed a 60-minute cutoff consistent with other studies assessing geographic accessibility to emergency services [75–79]. The purpose of using 50^th^, 75^th^ and 95^th^ percentile of the transit time distribution on the geographic accessibility measurement of the 2SFCA is that moving vehicles such as ambulances will cover shorter distances in the same total time, and thus service catchment areas will shrink with longer transit times due to congested traffic conditions (i.e., more minutes taken to cover 1km in Table 2).

## Results

### Spatial distribution of emergency health services

**Fig 1:**
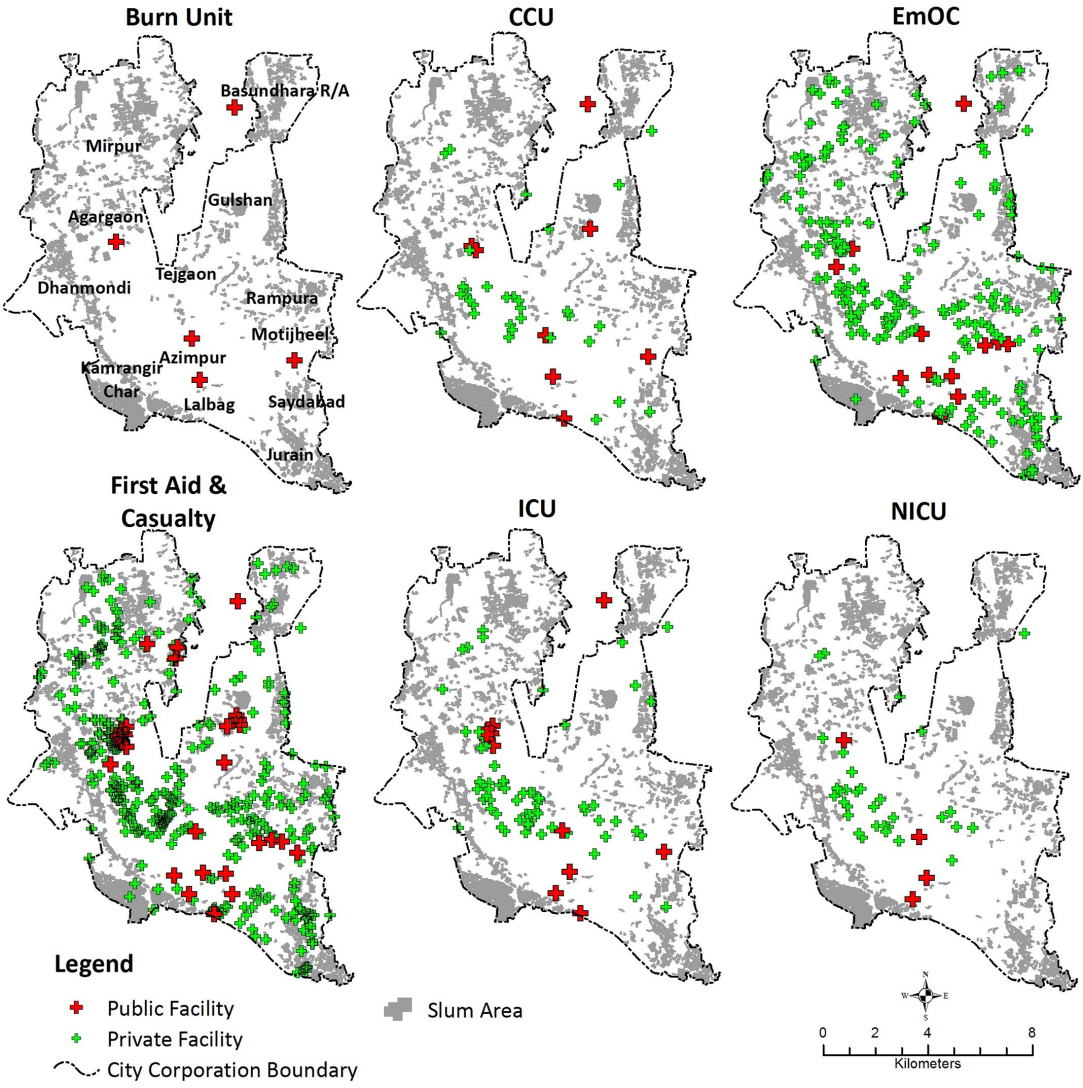
Distribution of emergency healthcare service providers and slums in Dhaka City.

As shown in Fig 1, the large number of EmOC and First Aid & Casualty providers is fairly evenly distributed within Dhaka’s boundaries. Remaining emergency services are generally concentrated in the central part of Dhaka City where government offices, universities and parks concentrate in the areas surrounding Azimpur, between Tejgaon in the north and Lalbag in the south, and between Dhanmondi and Rampura. These areas are comparatively less densely populated than the older and well-established southern areas of the city (Lalbag), as also indicated by the low density of slums. Conversely, Burn Units, Neonatal Care Units (NICU), Coronary Care Units (CCU), and Intensive Care Units (ICU) are less concentrated in peripheral areas, characterized instead by significant density of slums, such as the northern (Mirpur) and south-eastern (Rampura, Saydabad and Jurain) parts of the city. EmOC and First Aid & Casualty services are more uniformly distributed across the city area. In the following section we first discuss results pertaining to the geographic coverage and accessibility of emergency services to the slum population in moderate traffic conditions (i.e. 50^th^ percentile of transit time). We then present results in the context of increasingly congested traffic conditions (i.e. 75^th^ and 95^th^ percentiles of transit time) on the geographic coverage and accessibility.

### Geographic accessibility of slum settlements to emergency services in moderate traffic conditions

**Fig 2:**
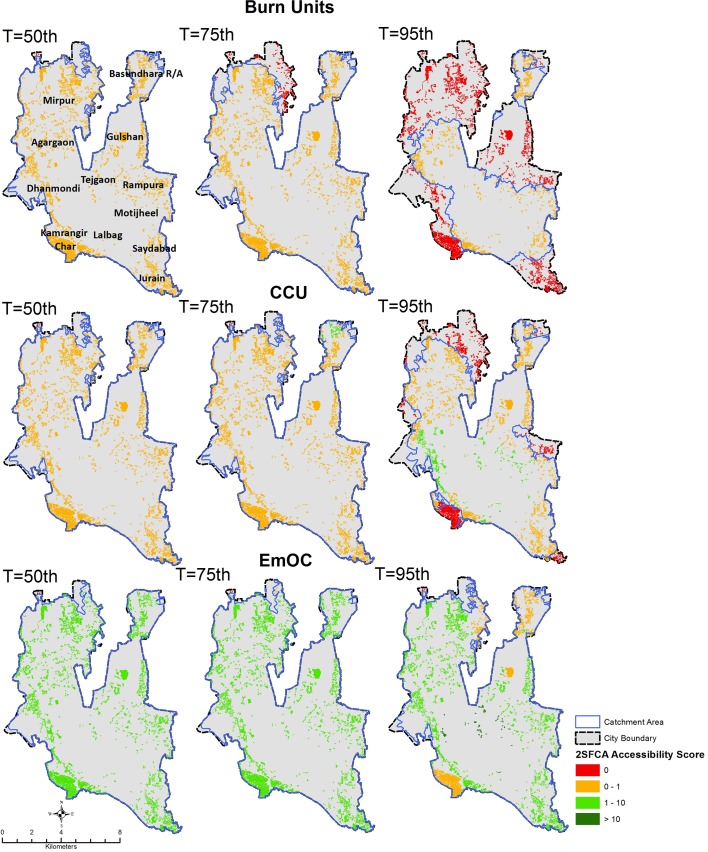
Geographic accessibility to Burn Unit, CCU, and EmOC emergency units in Dhaka city using a 60-minutes travel time threshold and different traffic scenarios characterized by transit time percentiles (T). Slums are color coded based on the spatial accessibility scores output of the 2-step floating catchment area method, expressed in units of nr of emergency units per 50,000 population. Blue polygons indicate the merged extent of the 60-minutes catchment areas of individual services (note that catchment areas are clipped to City Corporation extent).

**Fig 3:**
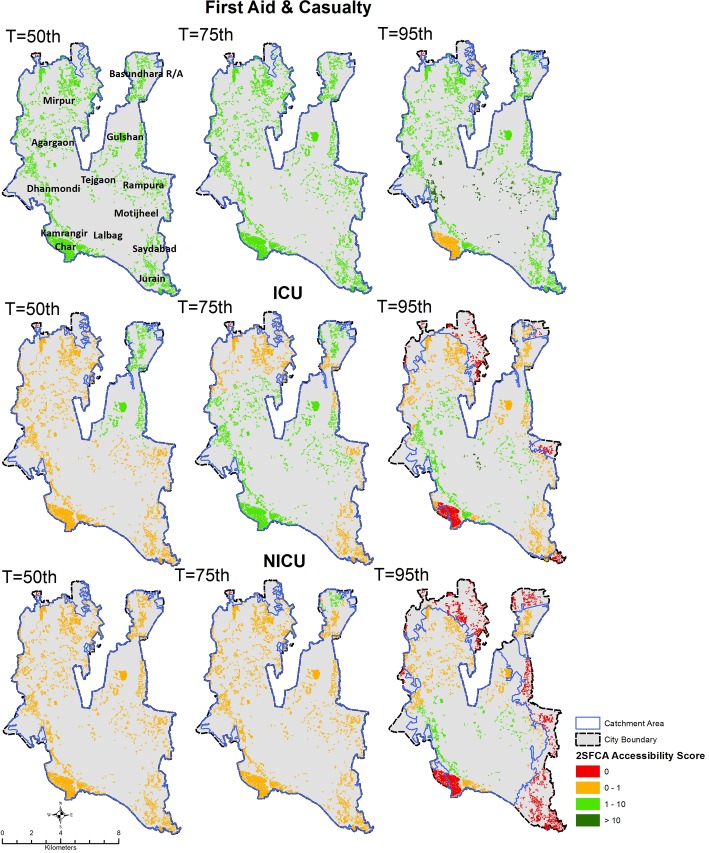
Geographic accessibility to First Aid & Casualty, ICU, and NICU emergency units in Dhaka city using a 60-minutes travel time threshold and different traffic scenarios characterized by different transit times percentiles (T). Slums are color coded based on the spatial accessibility scores output of the 2-step floating catchment area method, expressed in units of nr of emergency units per 50,000 population. Blue polygons indicate the merged extent of the 60-minutes catchment areas of individual services (note that catchment areas are clipped to City Corporation extent).

**Fig 4:**
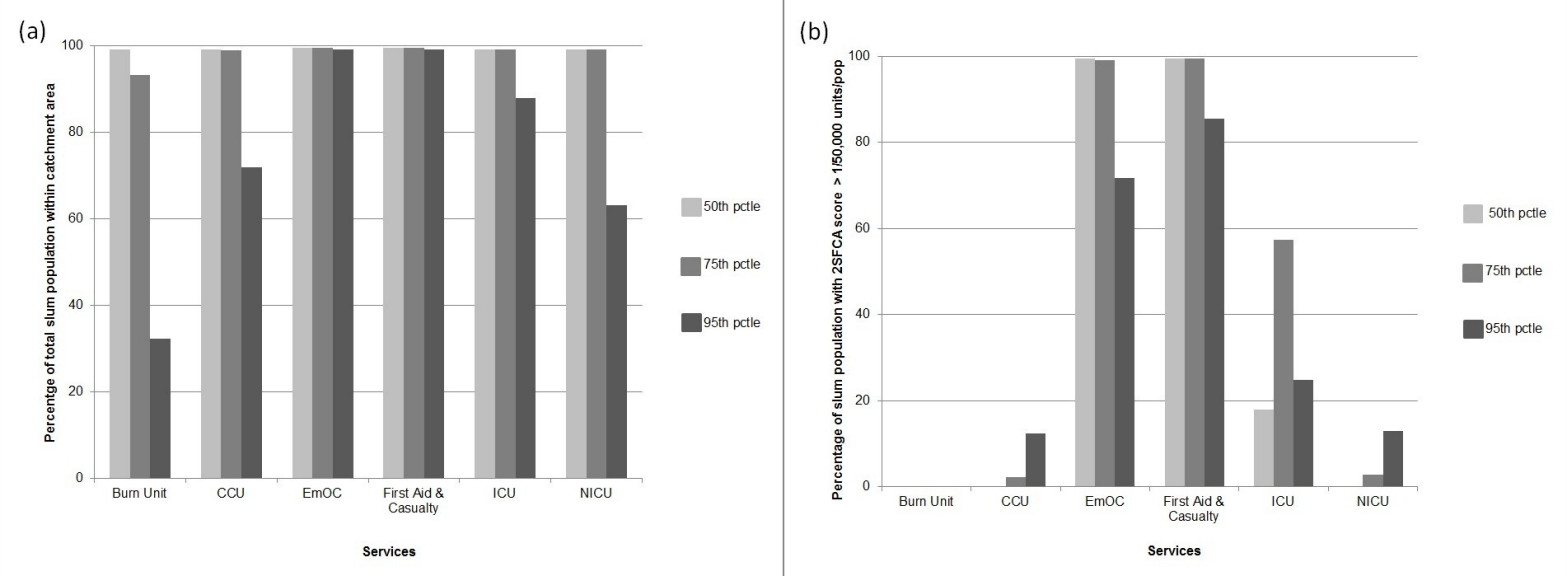
(a) Percentage of slum population within emergency unit catchment area and (b) Percentage of slum population with 2SFCA accessibility score above the threshold of 1 emergency units per 50,000 population, for different traffic scenarios (50^th^, 75^th^ and 95^th^ percentile of transit times distribution) and assuming 60 minute travel time threshold.

Figs 2 and 3 display the results of the geographic accessibility analysis of slum settlements to emergency services when considering 60-minutes travel time. In Fig 4 the same results are quantified in terms of the percentage of the total slum population in Dhaka city having geographic coverage and accessibility to each type of service.

When considering moderate traffic conditions (50^th^ percentile transit time scenario), the majority of slum population in Dhaka City fall within 60-minutes travel time from all emergency services considered, indicating a fairly comprehensive geographic coverage. In other words, all services are reachable within 60-minutes by nearly 100% of the slum population. However, when looking at the results in terms of the accessibility score output of the 2SFCA method, a larger variability of access to emergency care emerges between different types of services as a result of weighting supply and demand of services. Overall, the percentage of slum population having an accessibility score of at least 1 emergency unit per 50,000 population (Fig 4B) is, as expected, lower than the proportion of slum population within the 60-minutes catchment area. For example, in moderate traffic conditions, 90% of slum population is within 60-minutes reach from a Burn Unit, however, not a single slum has an accessibility score of at least 1 emergency unit per 50,000 population.

As observed for geographic coverage, geographic accessibility is elevated for First Aid and EmOC services, with 100% of slum population having access to at least 1 emergency unit per 50,000 population for a 60-minutes travel time threshold. In the case of the remaining less numerous emergency services such as ICU, Burn Unit, CCU and NICU, only a fraction of the slum population has access to at least 1 emergency unit per 50,000 population (17% for ICU, and 0% for Burn Units, CCU and NICU), despite the majority of slums being within 60-minutes travel time from such services.

These results also show how the number and spatial distribution of emergency services play an important role in determining geographic accessibility. Burn Unit has the lowest accessibility because of their limited numbers. These services are publicly provided. By contrast, EmOC and First Aid & casualty see a strong presence of private sector providers, ensuring better geographic coverage and accessibility.

### Impact of varying traffic conditions on geographic accessibility to emergency services

Figs 2, 3 and 4 display variations in geographic coverage and geographic accessibility in terms of increasingly congested traffic conditions. This is simulated by mapping the catchment areas and 2SFCA spatial accessibility score using 60-minutes travel time threshold, and using the 75^th^ and 95^th^ percentile of the transit time distribution, corresponding to congested and heavily congested traffic conditions.

When looking at geographic coverage, increasing traffic congestion has a stronger impact for less numerous emergency services (Burn Unit, CCU, ICU and NICU). Geographic coverage decreases by an average of 23 percentage points across all emergency services when moving from moderate to very congested traffic conditions. The strongest impact was on the coverage of Burn Units (from 99% of total slum population within 60-minutes reach for moderate traffic conditions to only 32% for heavily congested conditions, a factor of 3).NICU, CCU and ICU are also impacted in heavily congested conditions, with a decrease of coverage to 63%, 70% and 87% of total slum population respectively. Therefore, for heavily congested traffic conditions, only 63% of slums in Dhaka City Corporation are within 60-minutes reach of most emergency services, and only 32% are within reach of a Burn Unit. The geographic coverage provided by EmOC and First Aid are largely unaffected by traffic congestion, with nearly 100% of slum population located within 60-minutes reach even in heavily congested traffic conditions.

Geographic accessibility also generally decreases with increasing traffic congestion for most services. However, this effect is less dramatic, with an average decrease in geographic accessibility of only 1.5 percentage points across all emergency services (compared to a 23 percentage point decrease for coverage). The decrease in geographic accessibility is also more apparent for widespread services such as EmOC and First Aid, with a decrease in the percentage of slum population having access to at least 1 emergency unit per 50,000 population of between 27 and 13 percentage points when moving from moderate to heavily congested traffic scenario (see Fig 4B). Conversely, for Burn Units, ICU, NICU and CCU this change is less than 12 percentage points. For heavily congested traffic conditions only 12% of slums in Dhaka City Corporation have accessibility to at least 1 emergency unit per 50,000 population for most emergency services (except Burn Units), and 0% have adequate access to Burn Units.

The visual display of changes in spatial patterns in Figs 2 and 3 helps assess how the observed decrease in geographic coverage and accessibility of slum population to emergency services due to increasing traffic congestion impacts different parts of the city. The decrease in geographic accessibility is seen to be quite heterogeneous across the city, albeit slightly more concentrated in peripheral areas of the city (northern-eastern part of Mirpur, Bashundhara, Rampura, Saydabad, Kamrangir Char and Jurain). Interestingly, patterns in the 2SFCA accessibility score also reveal areas where worsening traffic congestion leads to an increase in geographic accessibility to emergency services, despite the shrinking catchment areas. This is notable for clusters of slums in the central part of the city, such as areas surrounding Azimpur, between Tejgaon in the north and Lalbag in the south, and between Dhanmondi and Rampura, where most emergency services are concentrated (see Fig 1). For these slums, accessibility to emergency units per unit of slum population is higher in congested rather than moderate traffic conditions. This is likely due to a greater supply of emergency services per unit population for slums located in the vicinity of emergency services as catchment areas shrink with increasing traffic congestion. This is reflected in the slight increase in geographic accessibility observed in Fig 4B for CCU, ICU and NICU.

Figs 2 and 3 also identify the areas of Dhaka City in which congested traffic conditions will limit accessibility to emergency services. These include peripheral areas in the North (Mirpur and Basundhara) the South-Eastern parts of the city (Rampura, Saydabad, Jurain), and the South-Western area of Kamragirchar.

## Discussion

This paper uses GIS analysis to explore geographic coverage (i.e., timely access to care) and accessibility (i.e., adequate supply of healthcare per population) to emergency healthcare in the growing megacity of Dhaka, with a particular focus on slum settlements, and the impacts of traffic congestion. Across all the emergency services in this analysis, the coverage provided by Burn Unit was the lowest as only a few public sector facilities were equipped to provide these specialized services (Table 1). Unlike the United States (US), where 40% percent of the population has access to a burn centre within 60-minutes [80], under the most congested traffic conditions in Dhaka, 32% of slums were within a 60 minute reach of a Burn Unit, and not a single slum possessed the requisite 1 emergency unit per 50,000 population. This translates into about 3 million slum dwellers without timely access to emergency services for burn injury, and over 4 million without access to sufficient emergency health resources in intense traffic conditions. NICU, CCU and ICU service providers show better geographic coverage, but are still only providing timely coverage to respectively 63%, 71% and 87% of slum population in intense traffic conditions, and adequate supply to not more than 12% of Dhaka’s slum population. This compares to studies in the US and Canada which estimate that 80% of the population have spatial access to a tertiary trauma centre within 1 hour [81, 82].

In this analysis, Emergency Obstetric Care and First Aid and Casualty were found to provide timely access within 60-minutes to nearly 100% of the slum population, and adequate supply to more than 70% slum population under any traffic condition, owing to their high number and even distribution throughout the city. Similar results indicating good geographic coverage of EmOC services within 60-minutes travel time were also found in another city in Bangladesh [52], although the country wide scenario is less optimistic, with only 41% of catchment population having access to Basic Emergency Obstetric and Newborn Care (BEmONC) within 1 hour [48]. The fact that Bangladesh has a crisis of health workforce [83] may be a reason why highly specialized care such as CCU and ICU are so scarce even in the capital city. A prior census and survey of health facilities in Dhaka City, showed that gynecologists and obstetricians comprise the highest share of specialist doctors in Dhaka City (Health Facility Mapping in Dhaka, Chittagong, & Khulna City Corporations, Bangladesh, Unpublished), possibly supporting the higher availability of EmOC care. Given the scale of global investments in decreasing maternal and child mortality in low- and middle-income countries that rank only second to HIV [84], the availability and geographic spread of these services is not surprising.

Inequities in access to emergency healthcare were also revealed. This is partially the result of the uneven distribution of health facilities themselves, but also to the intense traffic conditions experienced in many urban settings. Increasing traffic congestion was found to amplify inequities in geographic accessibility to emergency services by decreasing geographic accessibility to emergency services of population in peripheral slums located further away from available emergency services, while increasing accessibility for slums located nearer the city center or in older neighborhoods where emergency health facilities tend to concentrate. By implication, it is likely that providers in peripheral areas may be comparatively overburdened, and slum populations living in these areas are obliged to travel longer distances for treatment seeking than those located in more central parts of the city.

Spatial analysis also allowed the identification of specific slum clusters which have no or limited access within 60-minutes to most emergency services, particularly in the case of Burn Unit, NICU, ICU and CCU. These areas, namely Mirpur, Kamrangir Char, Jurain, Saydabad, Basundhara etc., are either located in the city periphery, or have been recently annexed to Dhaka City. In both instances, private and public sector providers are insufficient relative to population need. These areas should therefore be prioritized in expansion efforts that aim to increase healthcare coverage in Dhaka City.

Findings also shed light on the importance of considering traffic variability in measurements of geographic coverage and accessibility, particularly in urban settings. Recognizing this reality, a recent paper has modeled the importance of taking travel time into account in efforts to optimize service utilization in urban areas [85]. In this analysis, we also consider the impact of traffic variability on access to emergency care in Dhaka City. Our results indicate that the general practice of ignoring variability in traffic conditions [7, 29, 32, 39, 49, 70–72] significantly impacts the measurement of geographic coverage. We calculated up to a 3-fold overestimation of geographic coverage for sparsely distributed and less available services such as Burn Units, and a slightly lesser overestimation of geographic accessibility (1.4 times) for widely available services such as EmOC and First Aid and Casualty. Results further demonstrate that traffic congestion contributes to increasing inequitable access to services, by overestimating the geographic accessibility scores of peripheral areas and underestimating the same in central city areas. As a consequence, ignoring traffic congestion may lead to an overall overestimation of coverage and accessibility and an underestimation of intra-urban inequities in access to care. Traffic variability data, therefore, should be taken into account in service utilization research and urban health systems planning.

Traffic variability related to traffic congestion may be particularly critical in the event of serious accidents in which survival depends on timely access to healthcare [28]. The fatality rate due to road and pedestrian accidents in Bangladesh is very high and underscores the need to improve the accessibility of emergency services [86]. This is especially urgent in Dhaka, where in 2017, 22% of accident victims lost their lives due to delays in service utilization resulting from traffic congestion [87]. The situation is similar in neighboring India, where reports in 2016 suggest that slow traffic was related to the death of one third of trauma patients enroute to care [88]. In Karachi, Pakistan, traffic congestion and gridlocks were also found to hinder timely access to emergency medical services [89]. In addition to needless loss of life due to traffic congestion, the average speed of vehicles in Dhaka has reduced notably during last decade, around 66%, which translates into huge productivity losses in the range of 3 million working hours [90]. Moreover, as the Dhaka traffic survey indicates [74], these losses are heightened during peak morning hours, where the coefficient of variation of the transit time data is higher, indicating higher likelihood of congested traffic conditions.

Across our analysis, the persistent widespread coverage of EmOC and First Aid & Casualty is noteworthy. The majority of health facilities in Dhaka City are privately owned [91] and almost all of them provide EmOC and First Aid & Casualty care (Health Facility Mapping in Dhaka, Chittagong, & Khulna City Corporations, Bangladesh, Unpublished). Although limited in terms of other types of emergency service capacity, the substantial geographic reach of this sector, even in peripheral areas, emphasizes its significant role in urban settings [92]. However, while the geographic availability of private sector services may be greater, this does not make them, or any other private sector service, financially accessible to the urban poor. In health systems planning, therefore, it is imperative that the private sector be included, and at the same time, insurance or voucher systems are in place to ensure that these services are affordable.

Using GIS analysis, we portray a more realistic picture of the actual accessibility of emergency healthcare in Dhaka City of potential utility in managing a range of man-made and natural disasters and their health impacts [93]. Effective disaster response relies upon precise and related service information and socio-geographical mapping [94]. Beyond improving health service delivery planning, the approach we forward in may be useful in organizing emergency preparedness for pandemics, as well as natural or man-made disasters like fires which regularly occur in fast growing cities like Dhaka [95]. We envision that knowledge about the catchment area of emergency services will allow crisis command centres to act more efficiently. As urban centres continue to experience rapid growth, effective planning of health services is crucial in efforts to serve urban populations in an optimal fashion.

## Limitations of the study

The major limitation of the study was the lack of up-to-date slum data together with slum-specific population size. As a result, slum population had to be extrapolated from information on average slum population density and slum areal surface. The future availability of a more detailed slum census might improve the analysis presented. Also, as we could not differentiate between average transit times on secondary versus major roads. Instead, a constant transit time (in minutes per kilometer) was applied throughout the network for each traffic scenario. With heavier traffic (i.e. the 95^th^ percentile), however, we would expect secondary and tertiary roads to be as much or more congested as main roads. In this context, access to emergency services would be compromised even further. It should also be noted that the traffic data we used was collected by a GPS probed car, while slum dwellers usually use much slower rickshaws or Compressed Natural Gas (CNG) powered three wheelers. Were these differences taken into account the lack of timely access to emergency care would be even more dramatic.

## Conclusion

This study provides novel evidence on access to emergency services by slum dwellers in Dhaka City, and how variations in traffic conditions determine the degree to which services can be reached in a timely manner. As the successful management of critical patients requires that they reach emergency services quickly, geographic accessibility is paramount. Findings emphasize that it is not only the number but also the spatial distribution of key service points which are vital in maximizing access. It is recognized, however, the lack of health facility capacity data, a vital component for measuring access considered in several studies [96–98], hindered a more comprehensive calculation of accessibility. Nevertheless, the study provides compelling evidence of inadequate access to Burn Unit, CCU, NICU, and ICU services for slum dwellers in Dhaka. Moreover, our analysis identified specific slum clusters in peripheral areas which suffer poor accessibility to most emergency services. This calls for targeted interventions to improve access in these underserved areas. Any effort to improve the geographic coverage of emergency services, however, must also take into account issues of financial accessibility. Given the costs of private sector care, social protection schemes are crucial in making emergency services affordable to the urban poor. Finally, our findings highlight the need to consider variable traffic conditions when assessing geographic accessibility to emergency health services in the urban settings. The temporality of traffic conditions and their consequences for timely emergency care is ubiquitous in growing megacities in LMICs, and arguably in any large city affected by traffic congestion.

## References

[1] Munoz UH, Kallestal C. Geographical accessibility and spatial coverage modeling of the primary health care network in the Western Province of Rwanda. International journal of health geographics. 2012;11(1):40. 22984920 10.1186/1476-072X-11-40 PMC3517388

[2] Masters S, Bursteina R, Amofahb G, Abaogyec P, Kumara S, Hanlon M. Travel time to maternity care and its effect on utilization in rural Ghana: A multilevel analysis. Social Science & Medicine. 2013;93(147–154). 10.1016/j.socscimed.2013.06.012 23906132

[3] Mwaliko E, Downing R, O’Meara W, Chelagat D, Obala A, Downing T, et al “Not too far to walk”: the influence of distance on place of delivery in a western Kenya health demographic surveillance system. BMC Health Services Research 2014;14(212). 10.1186/1472-6963-14-212 PMC4036729 24884489

[4] Gabrysch S. The influences of distance on health facility delivery in rural Zambia: London School of Hygiene & Tropical Medicine; 2010.

[5] Zegeye K, Gebeyehu A, Melese T. The Role of Geographical Access in the Utilization of Institutional Delivery Service in Rural Jimma Horro District, Southwest Ethiopia. Primary Health Care. 2014;4(1). 10.4172/2167-1079.1000152

[6] Schoeps A, Gabrysch S, Niamba L, Sié A, Becher H. The Effect of Distance to Health-Care Facilities on Childhood Mortality in Rural Burkina Faso. American Journal of Epidemiology. 2011;173(5):492–8. 10.1093/aje/kwq386 21262911

[7] Wang F, Luo W. Assessing spatial and nonspatial factors for healthcare access: Towards an integrated approach to defining health professional shortage areas. Health and Place. 2005;11(2):131–46. 10.1016/j.healthplace.2004.02.003 15629681

[8] Ray N, Ebener S. AccessMod 3.0: computing geographic coverage and accessibility to health care services using anisotropic movement of patients. International journal of health geographics. 2008;7 10.1186/1476-072X-7-7 19087277 PMC2651127

[9] Kobusingye OC, Hyder AA, Bishai D, Joshipura M, Hicks ER, C M. Emergency Medical Services In: Jamison DT, Breman JG, Measham AR, Alleyne G, Claeson M, Evans DB, et al, editors. Disease Control Priorities in Developing Countries 2nd edition. Washington (DC): The International Bank for Reconstruction and Development / The World Bank; 2006.

[10] Razzak JA, Kellermann AL. Emergency medical care in developing countries: is it worthwhile? Bulletin of the World Health Organization. 2002;80(11). PMC2567674 12481213

[11] Muckart DJJ. Trauma—the malignant epidemic. S Afr Med J 1991;79:93–5. 1989097

[12] WHO. Background paper for the technical consultation on effective coverage of health systems 27–29 August 2001; Rio de Janeiro, Brazil. Geneva, Switzerland: WHO; 2001.

[13] Schonfeld HK, Heston JF, Falk IS. Numbers of physicians required for primary medical care. N Engl J Med. 1972;286. 10.1056/NEJM197203162861104 5058795

[14] Connor RA, Hillson SD, Krawelski JE. Competition, professional synergism, and the geographic distribution of rural physicians. Med Care. 1995;33. 10.1097/00005650-199511000-00001 7475417

[15] Haynes R, Bentham G, Lovett A, Gale S. Effects of distances to hospital and GP surgery on hospital inpatient episodes, controlling for needs and provision. Social Science and Medicine. 1999;49(3):425–33. 10.1016/s0277-9536(99)00149-5 10414825

[16] Gyimah S, Takyi B, Addai I. Challenges to the reproductive health needs of African women: on religion and maternal health utilization in Ghana. Social Science & Medicine. 2006;62(12):2930–44. 10.1016/j.socscimed.2005.11.034 16406206

[17] Schuurman N, Fiedler R, Grzybowski S, Grund D. Defining rational hospital catchments for non-urban areas based on travel-time. International journal of health geographics. 2006;5 10.1186/1476-072X-5-5 17018146 PMC1617091

[18] Guagliardo MF, Ronzio CR, Cheung I, Chacko E, Joseph JG. Physician accessibility: an urban case study of pediatric providers. Health & place. 2004;10(3):273–83. 15177201 10.1016/j.healthplace.2003.01.001

[19] Radke J, Mu L. Spatial decomposition, modeling and mapping service regions to predict access to social programs. Geographic Information Sciences. 2000;6:105–12.

[20] Joseph A, Bantock P. Measuring potential physical accessibility to general practitioners in rural areas: a method and case study. Social Science and Medicine. 1982;16(1):85–90. 10.1016/0277-9536(82)90428-2 7100960

[21] Luo W, Wang F. Measures of spatial accessibility to health care in a GIS environment: Synthesis and a case study in the Chicago region. Environment and Planning B: Planning and Design. 2003;30(6):865–84. 10.1068/b29120 PMC8238135 34188345

[22] Ye H, Kim H. Measuring Spatial Health Disparity Using a Network-Based Accessibility Index Method in a GIS Environment: A Case Study of Hillsborough County, Florida. International Journal of Geospatial and Environmental Research. 2014;Vol. 1: No. 1, Article 2.

[23] Luo W, Qi Y. An enhanced two-step floating catchment area (E2SFCA) method for measuring spatial accessibility to primary care physicians. Health Place. 2009;15:1100–7. 10.1016/j.healthplace.2009.06.002 19576837

[24] Luo W, Whippo T. Variable catchment sizes for the two-step floating catchment area (2SFCA) method. Health Place. 2012;18:789–95. 10.1016/j.healthplace.2012.04.002 22560115

[25] Kim Y, Byon Y-J, Yeo H. Enhancing healthcare accessibility measurements using GIS: A case study in Seoul, Korea. PLOS ONE. 2018;13(2):e0193013 10.1371/journal.pone.0193013 29462194 PMC5819796

[26] Brabyn L, Skelly C. Modeling population access to New Zealand public hospitals. International journal of health geographics. 2002;1 10.1186/1476-072X-1-1 12459048 PMC149398

[27] Christie S, Fone D. Equity of access to tertiary hospitals in Wales: A travel time analysis. Journal of Public Health Medicine. 2003;25(4):344–50. 14747594 10.1093/pubmed/fdg090

[28] Vanderschuren M, McKune D. Emergency care facility access in rural areas within the golden hour?: Western Cape case study. International journal of health geographics. 2015;14(1):1–8. 25595608 10.1186/1476-072X-14-5 PMC4305393

[29] Yerramilli S, Fonseca DG. Assessing Geographical Inaccessibility to Health Care: Using GIS Network Based Methods. Public Health Research. 2014;4(5):145–59.

[30] Magadi M, Diamond I, Rodrigues R. The determinants of delivery care in Kenya. Society of Biology. 2000; 47(3–4):164–88. 10.1080/19485565.2000.9989017 12055693

[31] Raghupathy S. Education and the use of maternal health care in Thailand. Social Science & Medicine. 1996;43(4):459–71. 8844947 10.1016/0277-9536(95)00411-4

[32] McGrail MR. Spatial accessibility of primary health care utilising the two step floating catchment area method: an assessment of recent improvements. International journal of health geographics. 2012;11(1):50. 23153335 10.1186/1476-072X-11-50 PMC3520708

[33] Alegana VA, Wright JA, Pentrina U, Noor AM, Snow RW, Atkinson PM. Spatial modelling of healthcare utilisation for treatment of fever in Namibia. International journal of health geographics. 2012;11(1):6. 22336441 10.1186/1476-072X-11-6 PMC3292929

[34] Gething PW, Johnson FA, Frempong-Ainguah F, Nyarko P, Baschieri A, Aboagye P, et al Geographical access to care at birth in Ghana: a barrier to safe motherhood. BMC Public Health. 2012;12(1):991. 23158554 10.1186/1471-2458-12-991 PMC3533981

[35] Noor AM, Amin AA, Gething PW, Atkinson PM, Hay SI, Snow RW. Modelling distances travelled to government health services in Kenya. Tropical medicine & international health: TM & IH. 2006;11(2):188–96. 16451343 10.1111/j.1365-3156.2005.01555.x PMC2912494

[36] Okwaraji YB, Cousens S, Berhane Y, Mulholland K, Edmond K. Effect of Geographical Access to Health Facilities on Child Mortality in Rural Ethiopia: A Community Based Cross Sectional Study. PLoS ONE. 2012;7(3):e33564 10.1371/journal.pone.0033564 22428070 PMC3299799

[37] Mahmud SS, Hoque M, Bashir G, editors. Deficiencies of Existing Road Network in Dhaka Metropolitan City. Publication in 10th Pacific Regional Science Conference Organization (PRSCO) Summer Institute; 2008.

[38] Report Community, Zila Dhaka, Population and Housing Census 2011 Dhaka, Bangladesh: Bangladesh Bureau of Statistics; 2012.

[39] Cheng Y, Wang J, Rosenberg MW. Spatial access to residential care resources in Beijing, China. International journal of health geographics. 2012;11:32 10.1186/1476-072X-11-32 22877360 PMC3543173

[40] Yang D-H, Goerge R, Mullner R. Comparing GIS-Based Methods of Measuring Spatial Accessibility to Health Services. Journal of Medical Systems. 2006;30(1):23–32. 16548411 10.1007/s10916-006-7400-5

[41] McGrail M, Humphreys J. The index of rural access: an innovative integrated approach for measuring primary care access. BMC Health Serv Res. 2009;9:124 10.1186/1472-6963-9-124 19624859 PMC2720961

[42] McGrail MR, Humphreys JS. Measuring spatial accessibility to primary care in rural areas: improving the effectiveness of the two-step floating catchment area method. Appl Geogr. 2009;29.

[43] Dai D. Black residential segregation, disparities in spatial access to health care facilities, and late-stage breast cancer diagnosis in metropolitan Detroit. Health & Place. 2010;16. 10.1016/j.healthplace.2010.06.012 20630792

[44] Schuurman N, Berube M, Crooks VA. Measuring potential spatial access to primary health care physicians using a modified gravity model. Canadian Geographer. 2010;54.

[45] Wan N, Zhan FB, Zou B, Chow E. A relative spatial access assessment approach for analyzing potential spatial access to colorectal cancer services in Texas. Applied Geography. 2012;32(2):291–9.

[46] Houben R, Van Boeckel T, Mwinuka V, Mzumara P, Branson K, Linard C, et al Monitoring the impact of decentralised chronic care services on patient travel time in rural Africa—methods and results in Northern Malawi. International journal of health geographics. 2012;11(1):49. 23153311 10.1186/1476-072X-11-49 PMC3517381

[47] Rahman MH, Mosley WH, Ahmed S, Akhter HH. Does Service Accessibility Reduce Socioeconomic Differentials In Maternity Care Seeking? Evidence From Rural Bangladesh. Journal of Biosocial Science. 2008;40(01):19–33. 17588280 10.1017/S0021932007002258

[48] Chowdhury ME, Biswas TK, Rahman M, Pasha K, Hossain MA. Use of a geographic information system to assess accessibility to health facilities providing emergency obstetric and newborn care in Bangladesh. International Journal of Gynecology & Obstetrics. 2017;138(2):164–70. 28453863 10.1002/ijgo.12196

[49] Islam MS, Aktar S. Measuring physical accessibility to health facilities—a case study on Khulna City. World Health Popul. 2011;12(3):33–41. 21677527 10.12927/whp.2011.22195

[50] Apparicio P, Abdelmajid M, Riva M, Shearmur R. Comparing alternative approaches to measuring the geographical accessibility of urban health services: Distance types and aggregation-error issues. International journal of health geographics. 2008;7(1):7. 18282284 10.1186/1476-072X-7-7 PMC2265683

[51] McGuirk MA, Porell FW. Spatial Patterns of Hospital Utilization: The Impact of Distance and Time. Inquiry. 1984;21(1):84–95. 6232220

[52] Panciera R, Khan A, Rizvi SJR, Ahmed S, Ahmed T, Islam R, et al The influence of travel time on emergency obstetric care seeking behavior in the urban poor of Bangladesh: a GIS study. BMC Pregnancy and Childbirth. 2016;16(1):1–13. 27549156 10.1186/s12884-016-1032-7 PMC4994156

[53] Jahan NA, Howlader SR, Sultana N, Ishaq F, Sikder MZH, Rahman T. Health Care Seeking Behavior of Slum-Dwellers in Dhaka City Dhaka, Bangladesh: Institute of Health Economics, University of Dhaka, 2015.

[54] Baumgart S, Hackenbroch K, Hossain S, Hossain S. Urban Development and Public Health in Dhaka, Bangladesh In: Kramer A, Khan MH, Kraas F, editors. Health in Megacities and Urban Areas. Contributions to Statistics. Germany: Physica, Heidelberg; 2011 p. 281–300.

[55] Dhaka North City Corporation [cited 2016 18 July]. Available from: http://www.dncc.gov.bd/.

[56] Dhaka South City Corporation [cited 2016 18 July]. Available from: http://www.dhakasouthcity.gov.bd/.

[57] Dhaka Population [cited 2018 21 January]. Available from: http://worldpopulationreview.com/world-cities/dhaka-population/.

[58] United Nations DESA, Population Division. World Urbanization Prospects: The 2014 Revision, (ST/ESA/SER.A/366). 2015.

[59] Demographia World Urban Areas. 12th Annual Edition [Internet]. [cited 18 December, 2016]. Available from: www.demographia.com/db-worldua.pdf.

[60] European Commission Humanitarian Aid Office ADPC, Plan Bangladesh, and Islamic Relief Worldwide Bangladesh. Urban Risk Assessment: A Facilitator's Guidebook. 2010.

[61] Streatfield PK, Karar ZA. Population Challenges for Bangladesh in the Coming Decades. Journal of Health, Population, and Nutrition. 2008;26(3):261–72. 18831223 PMC2740702

[62] Gruebner O, Sachs J, Nockert A, Frings M, Khan MMH, Lakes T, et al Mapping the Slums of Dhaka from 2006 to 2010. Dataset Papers in Science. 2014;2014:1–7.

[63] Preliminary Report on Census of Slum Areas and Floating Population 2014. Dhaka, Bangladesh: Bangladesh Bureau of Statistics (BBS), 2015.

[64] World Urbanisation Prospects: The 2007 revision. Executive summary. Population Division of the Department of Economic and Social Affairs of the United Nations Secretariat (UNPD), 2009.

[65] Islam R, Zakaria R, Hasan M, Saha S, Ahmed R, Ahmed S, et al Health facility mapping in Rajshahi & Narayanganj city corporations, Bangladesh. Dhaka, Bangladesh: icddr,b, 2016.

[66] Mock C, Lormand J-D, Goosen a, Joshipura M, Peden M. Guidelines for essential trauma care Geneva: World Health Organization, 2004.

[67] MOLGRDC. Second Urban Primary Health Care Project. Bid Document: Project Management Unit (PMU), Local Government Division, Government of Bangladesh; 2005.

[68] Angeles G, Lance P, Barden-O'Fallon J, Islam N, Mahbub AQM, Nazem NI. The 2005 census and mapping of slums in Bangladesh: design, select results and application. International journal of health geographics. 2009;8(1):32. 19505333 10.1186/1476-072X-8-32 PMC2701942

[69] Mohit MA. Bastee Settlements of Dhaka City, Bangladesh: A Review of Policy Approaches and Challenges Ahead. Procedia—Social and Behavioral Sciences. 2012;36(Supplement C):611–22.

[70] Patel AB, Waters NM, Ghali WA. Determining geographic areas and populations with timely access to cardiac catheterization facilities for acute myocardial infarction care in Alberta, Canada. International journal of health geographics. 2007;6:47 10.1186/1476-072X-6-47 17939870 PMC2173884

[71] Higgs G. A Literature Review of the Use of GIS-Based Measures of Access to Health Care Services. Health Services and Outcomes Research Methodology. 2004;5(2):119–39.

[72] Haynes R, Jones AP, Sauerzapf V, Zhao H. Validation of travel times to hospital estimated by GIS. International journal of health geographics. 2006;5 10.1186/1476-072X-5-5 16984650 PMC1586189

[73] Banik B, Chowdhury M, Sarkar M. Study of the Traffic congestion in Sylhet City. Journal of the Indian Roads Congress. 2009.

[74] Anwar AHMM. A Study on Factors for Travel Time Variability in Dhaka City Corporation Area. Journal of Bangladesh Institute of Planners. 2010;3:53–64.

[75] Wei R, Clay Mann N, Dai M, Hsia RY. Injury-based Geographic Access to Trauma Centers. Academic Emergency Medicine. 2019;26(2):192–204. 10.1111/acem.13518 30019802

[76] Carr BG, Branas CC, Metlay JP, Sullivan AF, Camargo CA. Access to Emergency Care in the United States. Annals of emergency medicine. 2009;54(2):261–9. 10.1016/j.annemergmed.2008.11.016 19201059 PMC2728684

[77] Nicholl J, West J, Goodacre S, Turner J. The relationship between distance to hospital and patient mortality in emergencies: an observational study. Emergency Medicine Journal: EMJ. 2007;24(9):665–8. 10.1136/emj.2007.047654 17711952 PMC2464671

[78] Ravelli ACJ, Jager KJ, de Groot MH, Erwich J, Rijninks-van Driel GC, Tromp M, et al Travel time from home to hospital and adverse perinatal outcomes in women at term in the Netherlands. BJOG: An International Journal of Obstetrics & Gynaecology. 2011;118(4):457–65. 21138515 10.1111/j.1471-0528.2010.02816.x

[79] Una E, Chen S, Waldorf B. Healthcare Access in Indiana 2008 [cited 2015 28 October]. Available from: https://www.pcrd.purdue.edu/files/…/Healthcare-Access-in-Indiana.pdf.

[80] Klein MB, Kramer CB, Nelson J, Rivara FP, Gibran NS, Concannon T. Geographic Access to Burn Center Hospitals. JAMA: the journal of the American Medical Association. 2009;302(16):1774–81. 10.1001/jama.2009.1548 PMC3045670 19861669

[81] Branas CC, MacKenzie EJ, Williams JC, et al Access to trauma centers in the united states. JAMA. 2005;293(21):2626–33. 10.1001/jama.293.21.2626 15928284

[82] Hameed SM, Schuurman N, Razek T, Boone D, Van Heest R, Taulu T, et al Access to Trauma Systems in Canada. Journal of Trauma and Acute Care Surgery. 2010;69(6):1350–61. 10.1097/TA.0b013e3181e751f7 20838258

[83] Ahmed SM, Hossain MA, RajaChowdhury AM, Bhuiya AU. The health workforce crisis in Bangladesh: shortage, inappropriate skill-mix and inequitable distribution. Human Resources for Health. 2011;9(1):3. 21255446 10.1186/1478-4491-9-3 PMC3037300

[84] Dieleman J, Campbell M, Chapin A, Eldrenkamp E, Fan VY, Haakenstad A, et al Evolution and patterns of global health financing 1995–2014: development assistance for health, and government, prepaid private, and out-of-pocket health spending in 184 countries. The Lancet. 2017;389(10083):1981–2004. 10.1016/S0140-6736(17)30874-7 PMC5440770 28433256

[85] Smith CM, Fry H, Anderson C, Maguire H, Hayward AC. Optimising spatial accessibility to inform rationalisation of specialist health services. International journal of health geographics. 2017;16(1):15 10.1186/s12942-017-0088-6 28431545 PMC5399864

[86] Ahmed B. Contemporary Issues and Priorities in Addressing the Road Safety Problems of Dhaka Metropolitan Area, Bangladesh. Journal of Bangladesh Institute of Planners. 2013;6:103–18.

[87] Colossal loss. The Daily Star. 2018 March 25.

[88] George AA, Krishna A, Dias T, Vargheese AS, Divya RS, editors. Golden aid an emergency ambulance system. 2017 International Conference on Networks & Advances in Computational Technologies (NetACT); 2017 20–22 7 2017.

[89] Khalid M, Khalid NB. Emergency medical services and road congestion: deadly path to hospitals. Pakistan Journal of Public Health. 2017;7(3):127–8.

[90] A Modern Dhaka is Key to Bangladesh’s Upper-Middle Income Country Vision [press release]. The World Bank, July 19 2017.

[91] Adams AM, Ahmed S, Hasan SM, Islam R, Islam MR, Mehjabin N, et al Mapping the Urban Healthcare Landscape in 5 City Corporations, Bangladesh. Dhaka: icddr,b, 2015.

[92] Adams AM, Islam R, Ahmed T. Who serves the urban poor? A geospatial and descriptive analysis of health services in slum settlements in Dhaka, Bangladesh. Health policy and planning. 2015;30(suppl 1):i32–i45. 25759453 10.1093/heapol/czu094 PMC4353891

[93] Health Bulletin 2016. Mohakhali, Dhaka: MIS, DGHS, Ministry of Health and Family Welfare, Bangladesh

[94] Bremer R. Policy Development in Disaster Preparedness and Management: Lessons Learned from the January 2001 Earthquake in Gujarat, India. Prehospital and Disaster Medicine. 2012;18(4):372–84. 10.1017/s1049023x00001345 15310051

[95] Islam MZ, Hossain KM. Fire Hazards in Dhaka City: An Exploratory Study on Mitigation Measures. IOSR Journal of Environmental Science, Toxicology and Food Technology. 2018;12(5):46–56.

[96] Polo G, Acosta CM, Ferreira F, Dias RA. Location-Allocation and Accessibility Models for Improving the Spatial Planning of Public Health Services. PLOS ONE. 2015;10(3):e0119190 10.1371/journal.pone.0119190 25775411 PMC4361743

[97] Tang J-H, Chiu Y-H, Chiang P-H, Su M-D, Chan T-C. A flow-based statistical model integrating spatial and nonspatial dimensions to measure healthcare access. Health & Place. 2017;47:126–38. 28881229 10.1016/j.healthplace.2017.08.006

[98] Kiani B, Bagheri N, Tara A, Hoseini B, Hashtarkhani S, Tara M. Comparing potential spatial access with self-reported travel times and cost analysis to haemodialysis facilities in North-eastern Iran. Geospatial health. 2018;13(2). 10.4081/gh.2018.703 30451464

